# Supplementary material for: Interplay of cell–cell contacts and RhoA/MRTF‐A signaling regulates cardiomyocyte identity
Source: EMBO J. 2018 May 15;37(12):e98133. doi: 10.15252/embj.201798133 (PMC6003642; doi:10.15252/embj.201798133)
Supplement: Supplementary file 1 — Appendix [file EMBJ-37-e98133-s001.pdf]

Appendix

Interplay of cell-cell contacts and RhoA/MRTF signaling regulates cardiomyocytic identity

Tatjana Dorn, Jessica Haas, Elvira I. Parrotta, Dorota Zawada, Harold Ayetey, Gianluca Santamaria, Laura Iop, Elisa Mastantuono, Daniel Sinnecker, Alexander Goedel, Ralf Dirschinger, Ilaria My, Svenja Laue, Tarik Bozoglu, Christian Baarlink, Tilman Ziegler, Elisabeth Graf, Rabea Hinkel, Giovanni Cuda, Stefan Kääb, Andrew A. Grace, Robert Grosse, Christian Kupatt, Thomas Meitinger, Austin G. Smith, Karl-Ludwig Laugwitz, Alessandra Moretti

<u>**Content**</u>

Appendix Figure Legends

Appendix Figures S1-8

Appendix Tables S1-3

Appendix References

APPENDIX FIGURES LEGENDS

Appendix Figure S1. Generation and characterization of PKP2^{mut} iPSCs.

A The table summarizes clinical features and results of genetic screening of the ARVC (PKP2^{mut}) patient. The presence of major or minor diagnostic criteria according to the modified ARVC Task Force Criteria (Marcus *et al.*, 2010) is indicated. The total number of major and minor criteria is given in the next-to-last row; a definite diagnosis of ARVC requires the presence of 2 major or 1 major and 2 minor or 4 minor criteria.

B ARVC (PKP2^{mut}) patient harbors a p.V587Afs*655 heterozygous mutation in the PKP2 protein, caused by a T nucleotide deletion in exon 8, in position 1760 of the *PKP2* coding region, resulting in a shift of the reading frame and generation of premature translation termination at the amino acid position 655.

C-G Reprogramming of PKP2^{mut} patient skin keratinocytes to iPSCs. Representative images of skin keratinocytes, early and late passage iPSCs from PKP2^{mut} patient indicating that, after infection with Sendai viruses encoding for OCT4, SOX2, KLF4 and c-MYC, keratinocytes and freshly generated iPSCs express Sendai viral HN proteins, detected by immunofluorescence, while longer passaged iPSCs lack Sendai cell surface antigens. Scale bars, 100 µm (left and middle panels), 200 µm (right panels) (C). iPSCs express the pluripotency markers NANOG (red) and TRA1-81 (green). Scale bar, 100 µm (D). RT-PCR analysis confirms reactivation of endogenous pluripotency genes (E) and loss of Sendai viral transgenes in iPSCs (F). PSK indicates parental skin keratinocytes, iPSK infected parental skin keratinocytes (E, F). Karyotyping of PKP2^{mut}-iPSCs revealed normal karyotypes (G).

H-I Pluripotency assays in PKP2^{mut}-iPSCs. *In vitro* differentiation of iPSCs into ectoderm (NESTIN- and TUJ1-positive neuronal cells), mesoderm (cardiac troponin T-positive myocytes) and endoderm (Sox17-positive cells). Shown

are representative images from differentiated PKP2^{mut}-iPSCs. Scale bars, 100 µm (left and middle panels), 150 µm (right panels) (H). Teratoma assay for pluripotency. Teratomas containing all three germ layers were generated from PKP2^{mut}-iPSC line. Scale bar, 200 µm (I).

Appendix Figure S2. Induction of lineage tracing at E7.5 is specific and mG-marked cells in *WT1$^{CreERT2/+}$;R26$^{mTmG/+}$* are true proepicardial progenitors.

A Immunostaining of alpha smooth muscle actin (α-SMA, magenta), Vimentin (VIM, magenta), mG (green), and mT (red) in sections of the *Wt1$^{CreERT2/+}$;R26$^{mTmG/+}$* mouse hearts at P28 after tamoxifen treatment at embryonic day E7.5 (E7.5). Expression of mG was observed in proepicardium-derived lineages of α-SMA^{+} smooth muscle (upper panels) and VIM^{+} epicardial (middle panels) and cardiac fibroblasts (lower panels), as indicated by the white arrows. Scale bars, 25 µm. Regions specified by white boxes are shown in higher magnification panels on the right; scale bars, 10 µm.

B Top, schematic representation of control experimental setup with oil injection in *Isl1$^{MerCreMer/+}$;R26$^{mTmG/+}$* or *Wt1$^{CreERT2/+}$;R26$^{mTmG/+}$* mice at 7.5 and their analysis at postnatal day 28 (P28). Immunostaining of PLIN1 (magenta), cTNT (magenta), mG (green) and mT (red) in *Isl1$^{MerCreMer/+}$;R26$^{mTmG/+}$* (left) and *Wt1$^{CreERT2/+}$;R26$^{mTmG/+}$* (right) with oil injection at E7.5 shows no mG signal throughout the whole heart. Nuclei are stained with Hoechst 33258 (blue). Scale bars, 400 µm. Framed areas are shown in higher magnifications below (a-d). Representative areas of cTNT^{+} (magenta) CMs (a and c) and PLIN1^{+} (magenta) epicardial adipocytes (b and d) of *Isl1$^{MerCreMer/+}$;R26$^{mTmG/+}$* (a and b) and *Wt1$^{CreERT2/+}$;R26$^{mTmG/+}$* (c and d) mouse hearts. Scale bars, 25 µm. Labelling: ra, right atrium; rv, right ventricle; la, left atrium; lv, left ventricle.

Appendix Figure S3. No activation of Cre in the absence of tamoxifen in *Isl1*$^{MerCreMer/+}$;*R26*$^{mTmG/+}$ and *Wt1*$^{CreERT2/+}$;*R26*$^{mTmG/+}$ mouse embryos at E9.5.

Top, schematic representation of a control experimental setup with oil injection in *Isl1*$^{MerCreMer/+}$;*R26*$^{mTmG/+}$ and *Wt1*$^{CreERT2/+}$;*R26*$^{mTmG/+}$ mice at E7.5 and their analysis at E9.5. Immunostaining of cTNT (magenta), mG (green) and mT (red) in *Isl1*$^{MerCreMer/+}$;*R26*$^{mTmG/+}$ (left) and *Wt1*$^{CreERT2/+}$;*R26*$^{mTmG/+}$ (right) with oil injection at E7.5 shows no mG signal throughout the whole embryo. Nuclei are stained with Hoechst 33258 (blue). Scale bars, 100 µm. Framed areas are shown in higher magnification below (a-f). Representative areas of Isl1^{+} SHF progenitors (magenta) (a), Wt1^{+} PEO progenitors (b and e) and epicardial cells (d) (both magenta), and cTNT^{+} CMs (magenta) (c and f) in *Isl1*$^{MerCreMer/+}$;*R26*$^{mTmG/+}$ (a-c) and *Wt1*$^{CreERT2/+}$;*R26*$^{mTmG/+}$ (d-f) embryos. Scale bars, 25 µm. Arrowheads indicate Isl1^{+} progenitors in a and Wt1^{+} epicardial and proepicardial cells in b and e.

Appendix Figure S4. Isl1^{+}/Wt1^{+}-derived CMs are prone to adipogenesis in response to proadipogenic cues.

A Scheme of the experimental setup for testing susceptibility to adipogenesis of mouse ESC-derived Isl1^{+} clones.

B Representative immunofluorescence images of CMs derived from Isl1^{+}/Wt1^{+} (left) and Isl1^{+}/Wt1^{-} (right) clones after 14 days culture in adipogenic medium and stained with cTNT (green) and lipid marker LipidTOX (red). Nuclei are stained with Hoechst 33258 (blue). Scale bars, 25 µm.

C Summary of the percentage of cTNT^{+}/LipidTOX^{-} and cTNT^{+}/LipidTOX^{+} CMs derived from Isl1^{+}/Wt1^{+} and Isl1^{+}/Wt1^{-} clones.

Appendix Figure S5. Characterization of NLS-WT1 and PPARγ overexpression in wt CMs.

A Drawings depict the lentiviral constructs used for expression of nuclear-tagged form of WT1 (NLS-WT1) and the corresponding control GFP vector.

Immunofluorescence analysis of wt CMs 7 days after infection with GFP and NLS1-WT1 lentivirus demonstrates expression of nuclear WT1 (magenta) only in NLS-WT1 transduced GFP^+ cells, as indicated by arrows. Phalloidin marks F-actin (red). Nuclei are stained with Hoechst 33258 (blue). Scale bars, 25 µm.

B Drawings depict the lentiviral constructs used for PPARγ overexpression and the corresponding control GFP vector. Immunofluorescence analysis of wt CMs 7 days after infection with GFP and PPARγ lentivirus demonstrates PPARγ expression in PPARγ-transduced GFP^+ cells, as indicated by arrows. CMs are visualized with cTNT (red). Nuclei are stained with Hoechst 33258 (blue). Scale bars, 25 µm.

C qRT-PCR analysis of myocytic and adipocytic genes shows similar expression levels in wt CMs transduced with control GFP (black bars) and NLS-WT1 (blue bars) lentivirus over time in culture. Expression values are normalized to *GAPDH*; n=2.

D Immunostaining shows activation of CEBPα (red) expression in PPARγ (magenta) transduced wt CMs within 7 days. Phalloidin marks F-actin (green). Nuclei are stained with Hoechst 33258 (blue). Scale bars, 25 µm.

Appendix Figure S6. Validation of shRNA targeting WT1 and analysis of scramble-shRNA/PPARγ overexpressing CMs.

A qRT-PCR analysis of *WT1* expression reveals decreased *WT1* transcript in wt CMs 7d after infection with a lentiviral construct encoding a shRNA targeting WT1 (shWT1). n=4 ;*$P<0.01$ vs scramble; t-test.

B Left, Western blotting using an antibody directed against WT1 demonstrates reduced expression of WT1 protein (30 kDa) in total lysates of wt CMs 7 days after infection with shWT1 lentivirus. Actin is shown as a loading control. Right, densitometric readings reveal an almost 80% knock-down of WT1 in shWT1-infected wt CMs compared to scramble control. Values are expressed

as the integrals (area x mean density) of each band normalized to actin and relative to scramble; n=3; *P<0.05 vs scramble; t-test.

C Scrambled shRNA has no effect on PPARγ-induced conversion of wt CMs as shown by immunocytochemistry after lentiviral overexpression of PPARγ and scrambled shRNA (Scramble/PPARγOE) or PPARγ alone (PPARγOE) at 14 (upper panels) and 21 (lower panels) days following infection. Both constructs express GFP simultaneously. Representative images show GFP (green, left panels) and cTNT (blue) in conjunction with lipid stain ORO (red, right panels) in the same cells. Scale bars, 50 μm.

D qRT-PCR analysis of myocytic and adipocytic genes shows similar expression levels in PPARγOE or Scramble/PPARγOE conditions at the indicated time points; n=3 (PPARγOE) and n=4 (Scramble/PPARγOE).

Appendix Figure S7. Endogenous and forced expression of MRTF-A in $PKP2^{mut}$ and wt CMs.

A Immunofluorescence analysis of MRTF-A (red) in wt and $PKP2^{mut}$ CMs at 0d, 1d, and 5d of culture reveals similar pancellular expression pattern between the two groups. cTNT (green) marks CMs. Nuclei are stained with Hoechst 33258 (blue). Scale bars, 25 μm.

B $PKP2^{mut}$ CMs 14d after infection with doxycycline-inducible GFP-MRTF-A lentivirus and continuous doxycycline treatment demonstrate cytoplasmic and nuclear MRTF-A (red) and GFP (green) overexpression. CMs are stained with cTNT (magenta) and nuclei with Hoechst 33258 (blue). Scale bars, 25 μm.

Appendix Figure S8. Generation and characterization of $MYH10^{mut}$ iPSCs and CMs.

A The table summarizes clinical features and results of genetic screening of the ARVC ($MYH10^{mut}$) patient. The presence of 2 major and 2 minor diagnostic criteria according to the modified ARVC Task Force Criteria (Marcus *et al.*, 2010) fulfils the diagnosis of ARVC in the patient.

B ARVC ($MYH10^{mut}$) patient harbours a novel p.R577* heterozygous nonsense mutation in the MYH10 protein, caused by a C>T mutation in exon 15, in position 1729 of the *MYH10* coding region, resulting in generation of premature translation termination at the amino acid position 577.

C-F Reprogramming of $MYH10^{mut}$ patient skin keratinocytes to iPSCs. iPSCs express the pluripotency markers NANOG (red) and TRA1-81 (green) (C) and OCT4 (green) (left panels) and SOX2 (green) (right panels) (D) as shown by immunofluorescence analysis. Nuclei are stained with Hoechst 33258 (blue). Scale bars, 50 µm (C), 500 µm (D). RT-PCR analysis confirms loss of Sendai viral transgenes in iPSCs. PSK indicates parental skin keratinocytes, iPSK infected parental skin keratinocytes (E). Karyotyping of $MYH10^{mut}$-iPSCs revealed normal karyotypes (F).

G Teratoma assay for pluripotency. Teratomas containing all three germ layers were generated from $MYH10^{mut}$-iPSC line. Scale bar, 200 µm.

H Analysis of *MYH10* transcript by qRT-PCR reveals similar expression levels in wt and $MYH10^{mut}$ CMs. Expression values are normalized to *GAPDH*; n=3.

I Western blotting demonstrates reduced expression of non-myosin IIB (NMIIB) protein (encoded by the *MYH10* gene) in total lysate of $MYH10^{mut}$ CMs. Actin is shown as a loading control (lower panels). Densitometric readings for NMIIB bands reveals an almost 50% reduction in $MYH10^{mut}$ myocytic lysates compared to wt ones. Values are expressed as the integrals (area x mean density) of each band normalized to actin and relative to wt (right panel); n=2; *P<0.05 vs wt; t-test.

J Immunofluorescence analysis for DSP (red) indicates an interrupted, abnormal expression at the plasma membrane of $MYH10^{mut}$ CMs compared to wt cells. cTNT (green) marks CMs. Nuclei are stained with Hoechst 33258 (blue). Scale bars, 12.5 µm.

A

Patient	ARVC (PKP2mut)
Sex	Male
Clinical features on presentation	Palpitations, pre-syncope, non-sustained ventricular tachycardia
Familial history	None
Depolarization/conduction defects	Major
Repolarization	Major
Global/regional dysfunction and/or structural alterations	Major
Arrhythmias	Major
Total diagnostic criteria	4 major, 0 minor
Mutation	c.1760delT; p.V587Afs*655 in PKP2

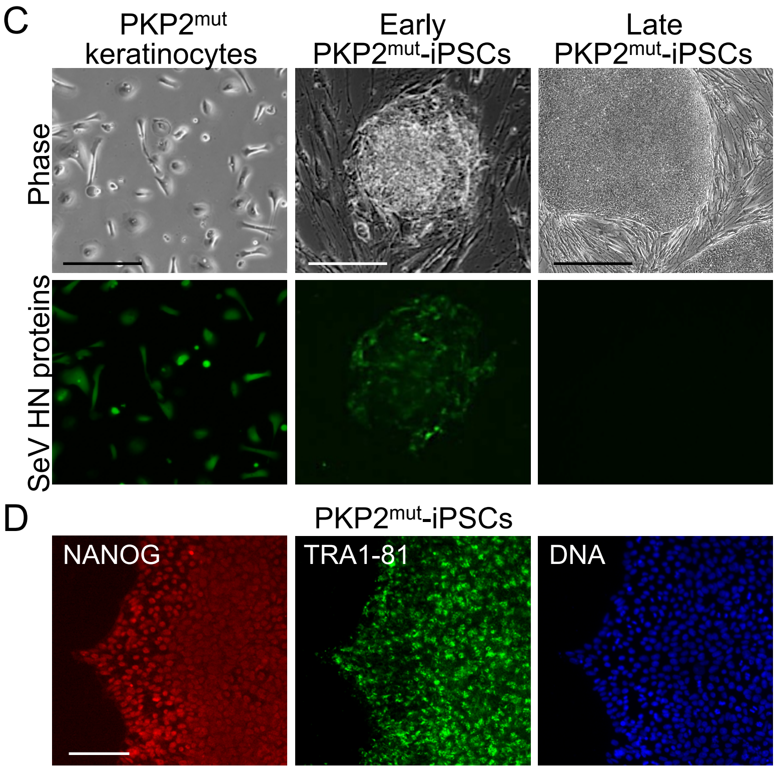

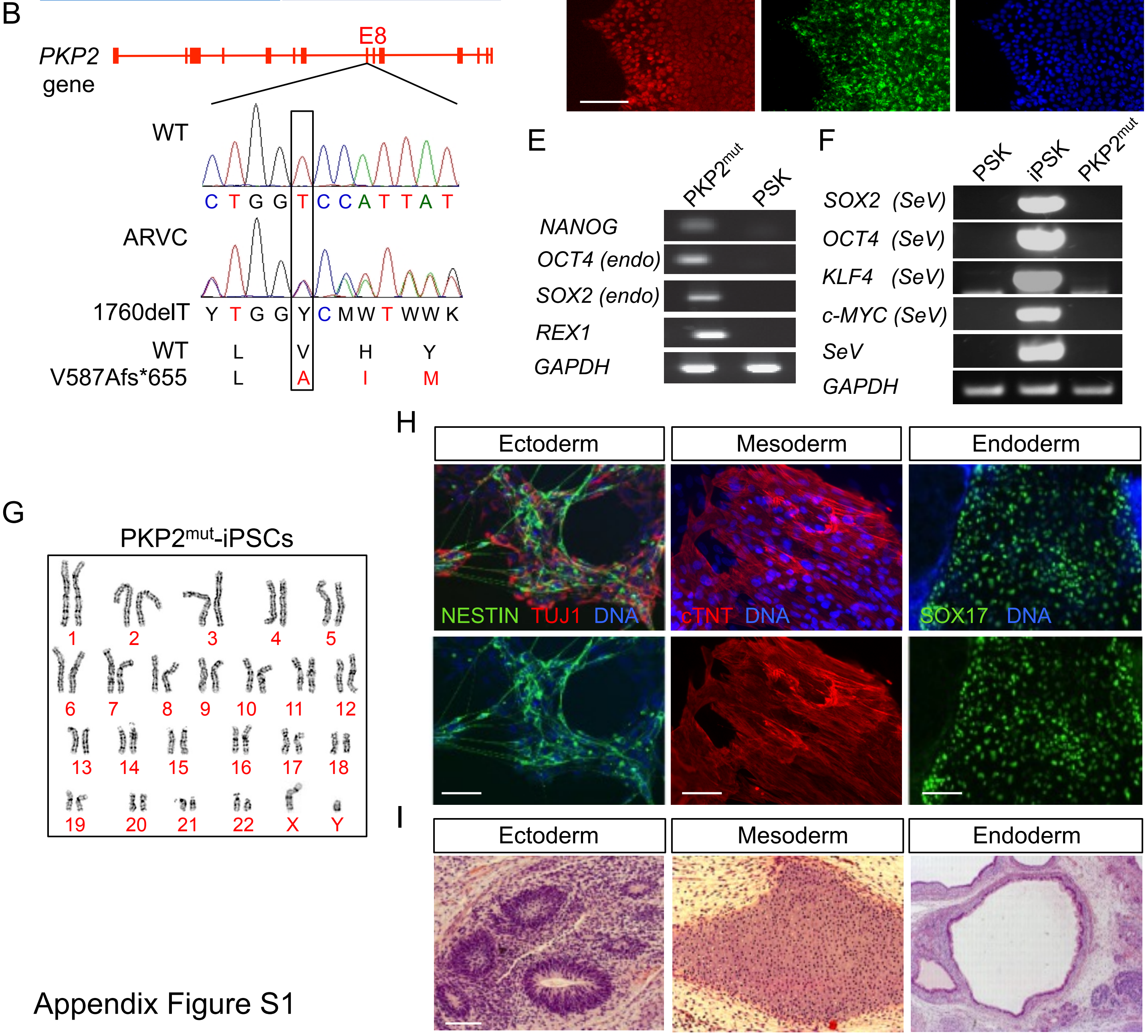

Appendix Figure S1

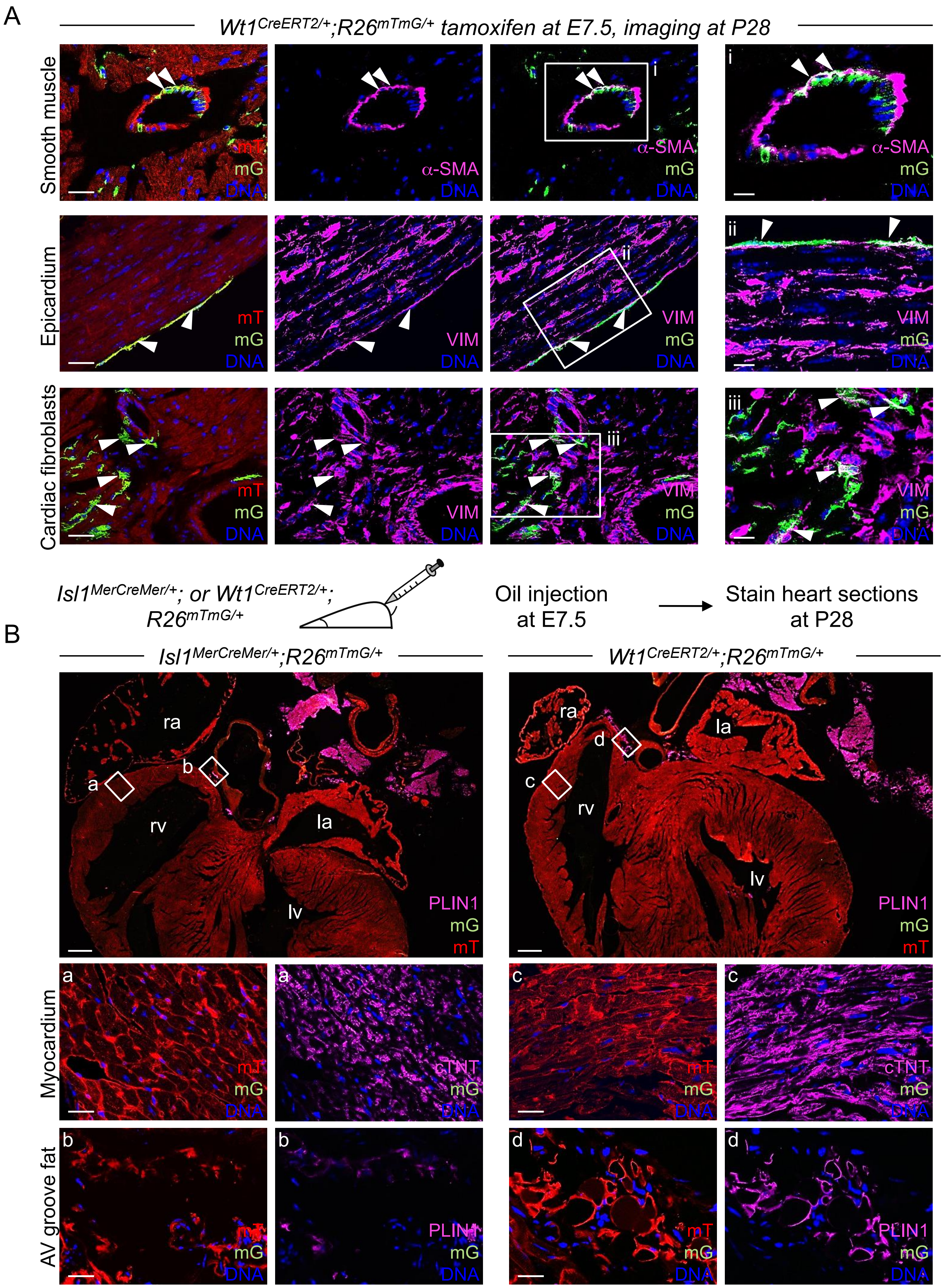

Appendix Figure S2

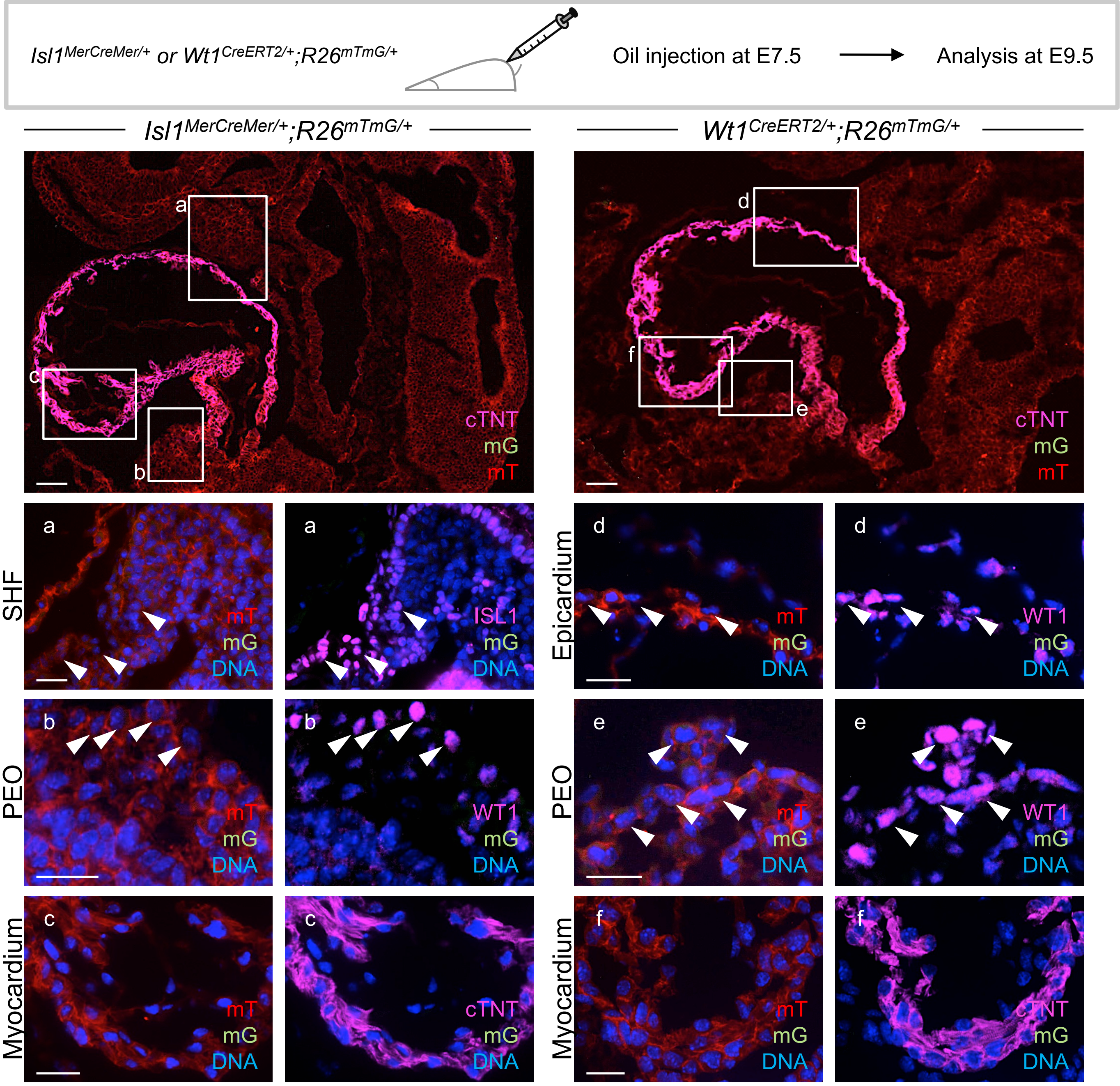

Appendix Figure S3

A

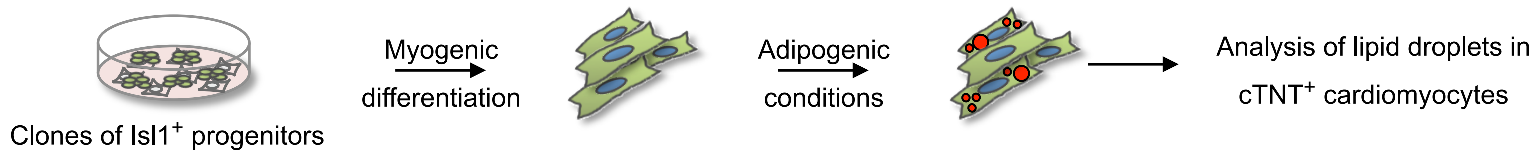

B

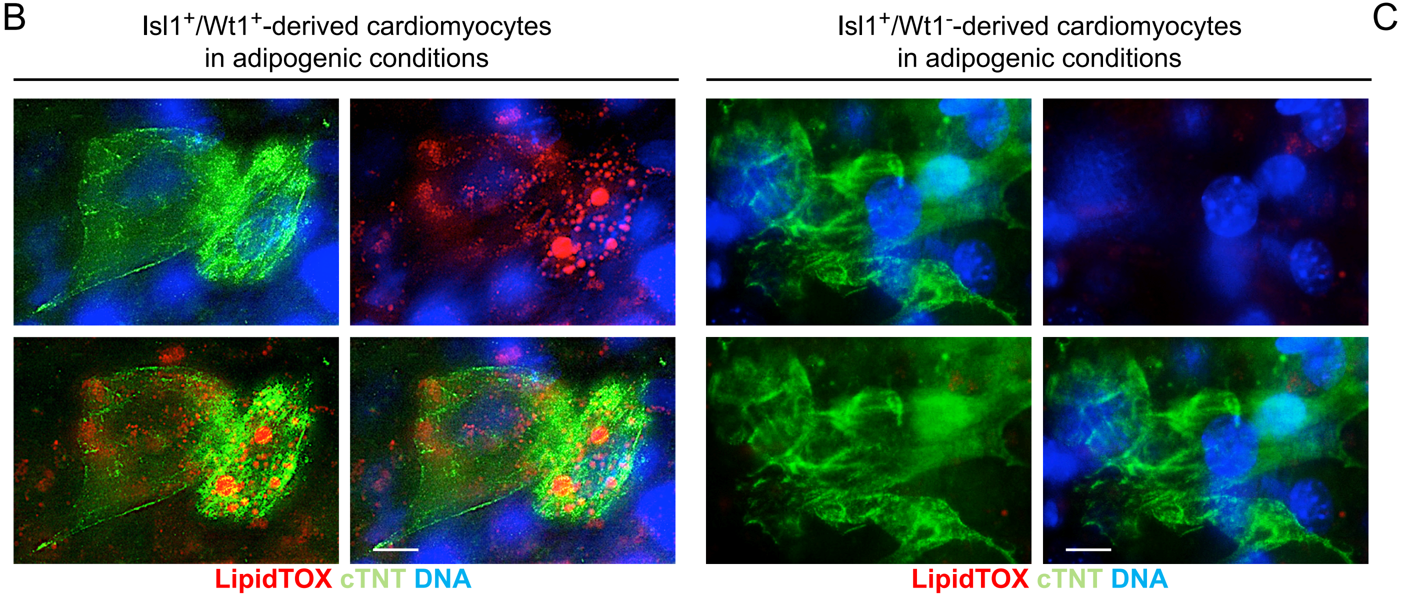

C

	Clone #	% of LipidTOX+/ cTNT+ CMs	% of LipidTOX−/ cTNT+ CMs
Isl1+/Wt1+	1	88	12
	2	83	17
	3	94	6
	4	58	42
	5	79	21
	6	33	67
	7	0	100
	**Mean ± SEM**	**62 ± 13**	**38 ± 13**
Isl1+/Wt1-	1	10	90
	2	0	100
	3	0	100
	4	5	95
	5	0	100
	**Mean ± SEM**	**3 ± 4**	**97 ± 4**

Appendix Figure S4

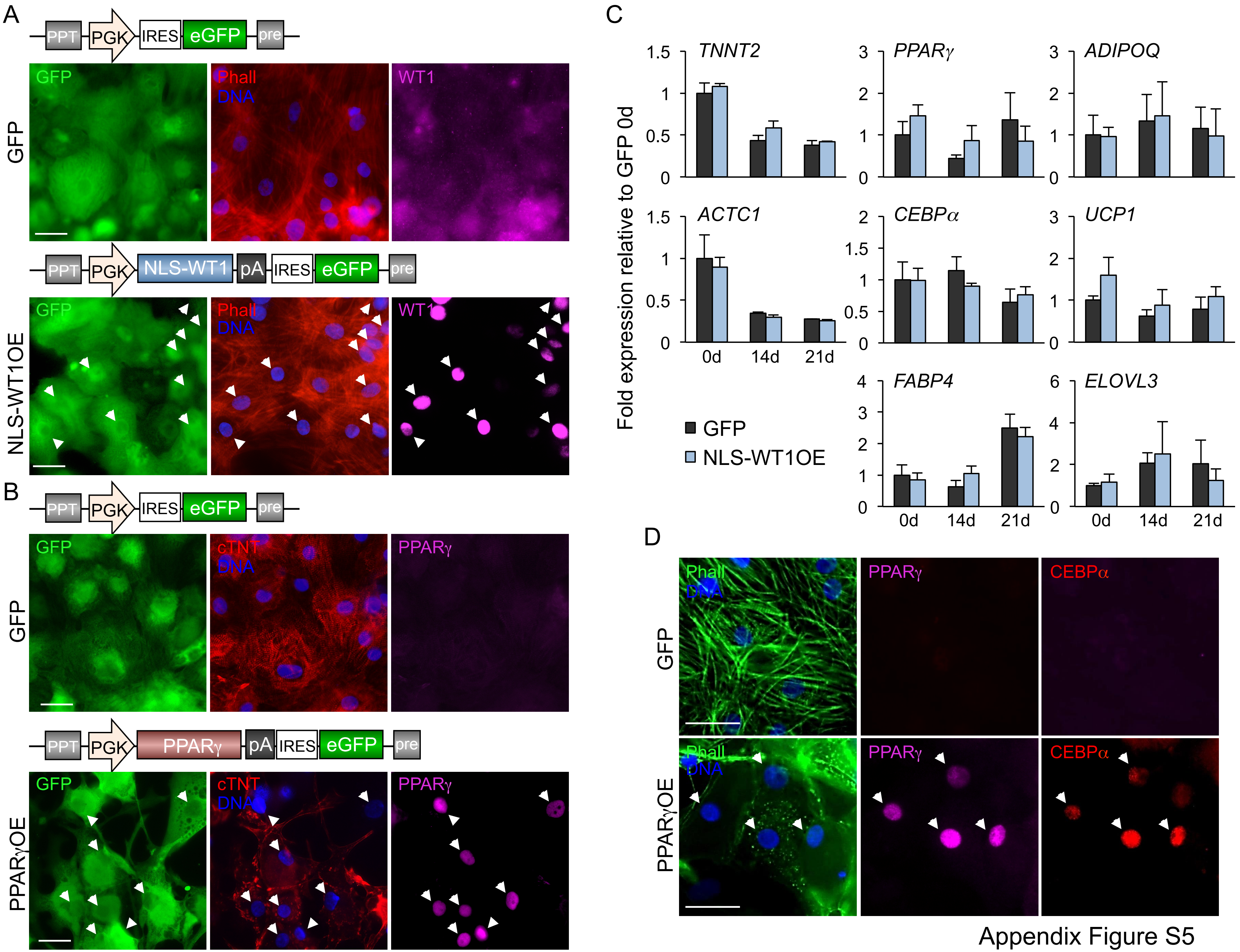

Appendix Figure S5

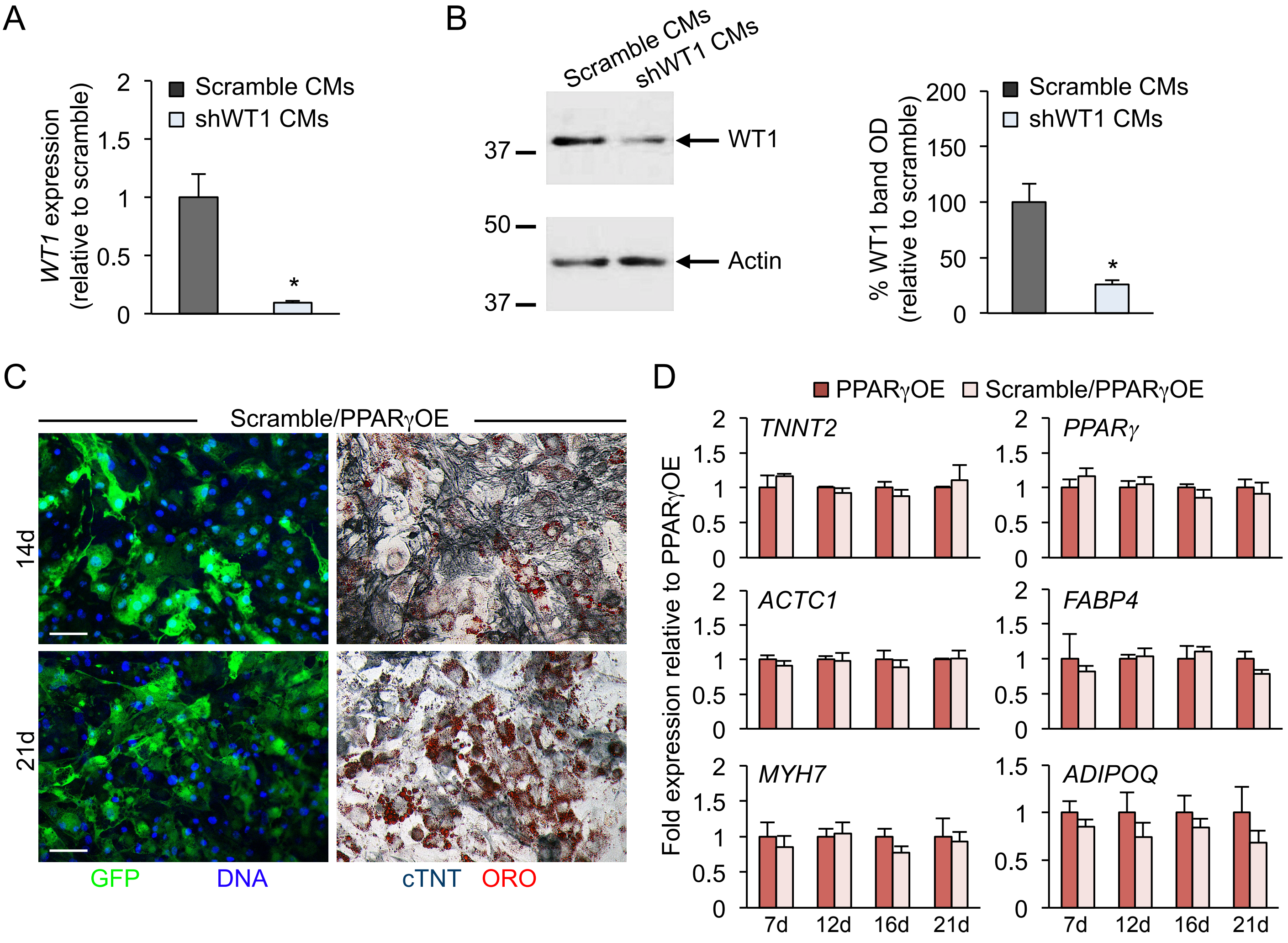

Appendix Figure S6

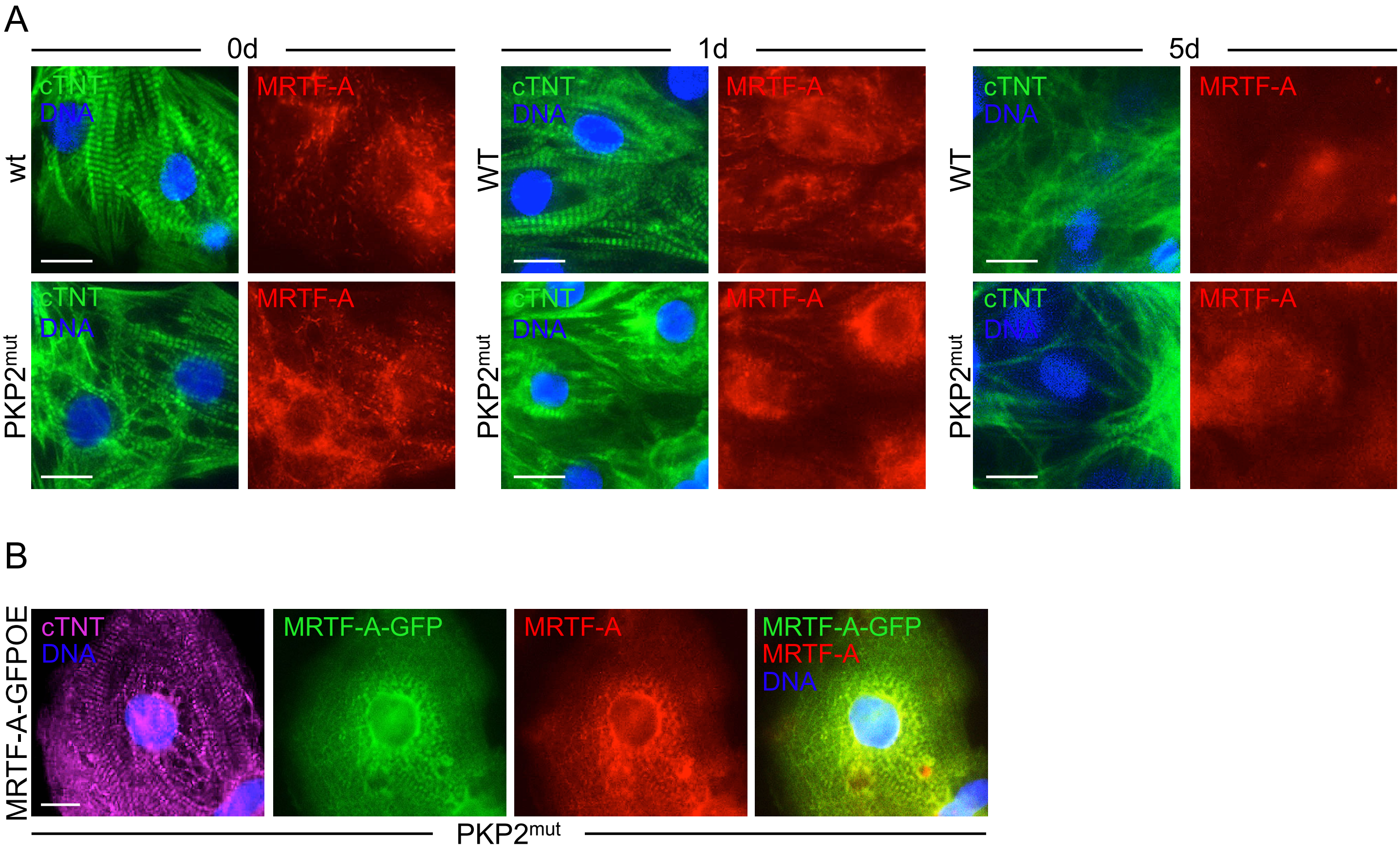

Appendix Figure S7

A

Patient	ARVC (MYH10^{mut})
Sex	Male
Clinical features on presentation	Ventricular flutter
Familial history	None
Depolarization/conduction defects	Major
Repolarization abnormalities	Minor
Global/regional dysfunction and/or structural alterations	Major
Arrhythmias	Minor
Total diagnostic criteria	2 major, 2 minor
Mutation	c.1729C>T, p.R577* in MYH10

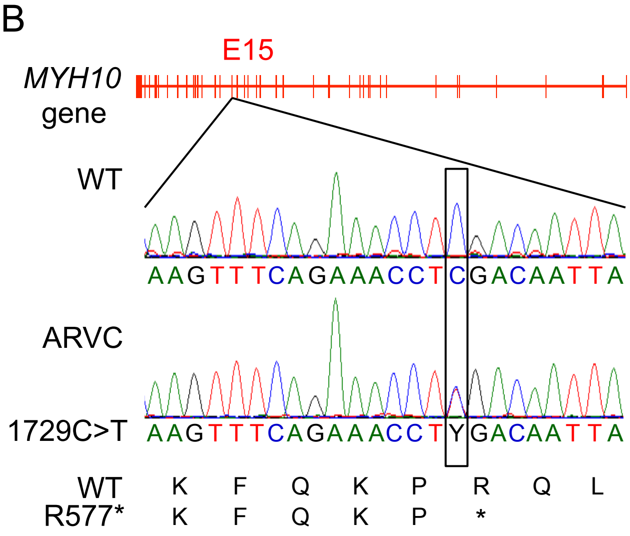

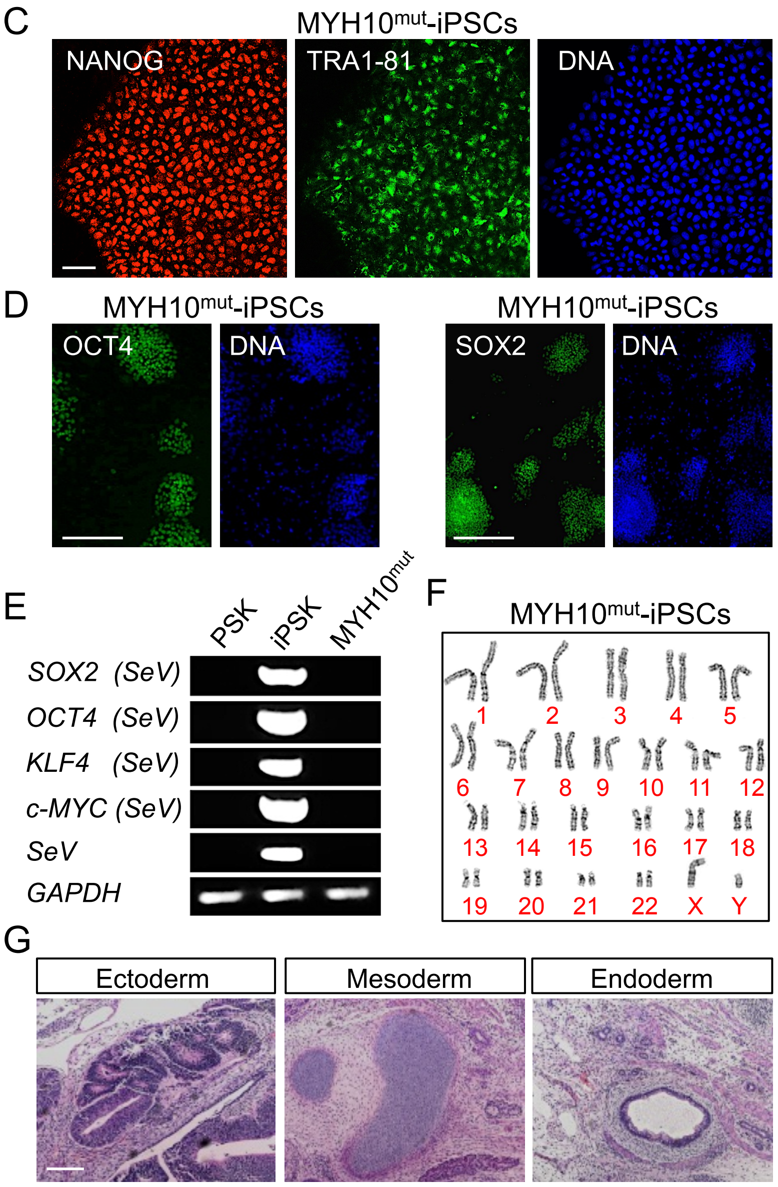

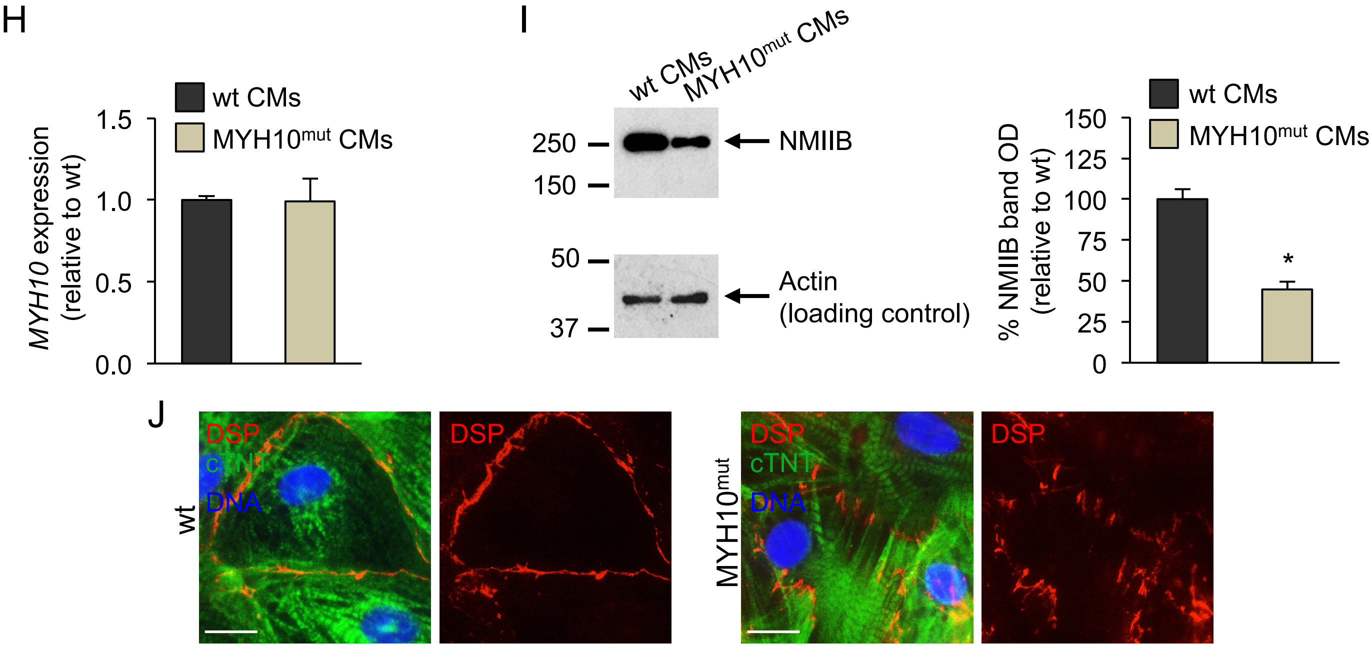

Appendix Figure S8

Appendix Table S1. List of ARVC-associated genes and their probabilities of loss of function intolerance (pLI).

Gene	Locus	ExAC pLI	ExAC n LOF expected	ExAC n LOF observed	Reference
PKP2	12p11	0	28.8	19	(Gerull *et al.*, 2004)
DSP	6p24.3	1	87.7	14	(Rampazzo *et al.*, 2002)
DSG2	18q12.1	0	26.1	10	(Pilichou *et al.*, 2006)
DSC2	18q12.1	0	25.9	10	(Syrris *et al.*, 2006)
JUP	17q21.2	0.04	19.1	6	(McKoy *et al.*, 2000)
CTNNA3	10q 22.2	0	68	11.7	(van Hengel *et al.*, 2013)
CDH2	18q12.1	0.9	29.1	5	(Mayosi *et al.*, 2017)
DES	2q35	0	16.7	7	(Klauke *et al.*, 2010)
TMEM43	3p25.1	0	17.2	13	(Merner *et al.*, 2008)
LMNA	1q22	0.99	19.1	1	(Quarta *et al.*, 2012)
TGFB3	14q24.3	0.92	12.3	1	(Beffagna *et al.*, 2005)
RYR2	1q43	1	164.5	27	(Tiso *et al.*, 2001)
PLN	6q22.1	0.11	1.2	1	(van der Zwaag *et al.*, 2012)
SCN5A	3p21	1	55.5	9	(Erkapic *et al.*, 2008)
TTN	2q32.1-q32.3	0	894	247	(Taylor *et al.*, 2011)
MYH10	17p13.1	1	79.9	10	

The table summarizes the probabilities of loss of function intolerance (pLI) calculated in the ExAC cohort considering the number of expected and observed LOF variants (canonical splice, frameshift and nonsense) for ARVC- associated genes and for the novel candidate *MYH10*. ExAC pLI score: probability of loss of function intolerance according to ExAC; ExAC n LOF expected: number of loss of function variants expected to be present in the gene in the ExAC cohort; EXAC n LOF observed: number of loss of function variants observed in the gene in the ExAC cohort.

Appendix Table S2. Primers and Taqman probes used for sequencing of the human *PKP2* (exon 8) and *MYH10* (exon 15) genes, RT-PCR, qRT-PCR, and ChIP-PCR as well as oligos for shRNA silencing

Human primers

Gene	Use	Primer forward	Primer reverse
ADIPOQ	qRT-PCR	GATGAAGTCCTGTCTTGGAAGG	CAGCACTTAGAGATGGAGTTGG
ACTC1	qRT-PCR	GCCCTGGATTTTGAGAATGA	ATGCCAGCAGATTCCATACC
BAX	qRT-PCR	CAAACTGGTGCTCAAGG	CCAACCACCCTGGTCTTGG
BIM	qRT-PCR	CCCTTTCTTGGCCCTTGT	CAGAAGGTTGCTTTGCCATT
CD137	qRT-PCR	AGATTTGCAGTCCCTGTCCTC	GAGGAACACTCCTTCCTGGTC
C-MYC (SeV-Tg)	RT-PCR	TAACTGACTAGCAGGCTTGTCG	TCCACATACAGTCCTGGATGATGATG
C/EBPα	qRT-PCR	GAAAGCTAGGTCGTGGGTCA	AGGCAATGCTGAAGGCATAC
CIDEA	qRT-PCR	CATGTATGAGATGTACTCCGTGTC	GAGTAGGACAGGAACCGCAG
DIO2	qRT-PCR	CCTCCTCGATGCCTACAAAC	GCTGGCAAAGTCAAGAAGGT
ELOVL3	qRT-PCR	ATGTAGTTCTGCCCCACAGC	AAGGACATGAGGCCCTTTTT
EPSTI1	qRT-PCR	AGGCAAAAGTCAACCAGGTG	TGAAGGCCAGATAGGAGTCAA
FABP4	qRT-PCR	AAAGAAGTAGGAGTGGGCTTTG	CTGATGATCATGTTAGGTTTGG
FAS	qRT-PCR	ATGCAGAAAGCACAGAAAGGA	CTTTCGAACAAAGCCTTTAACTTG
FBN1	qRT-PCR	TTCTGCAAGAGGATGGAAGG	GCAGTTGTGTTGCTTGGTTG
GAPDH	qRT-PCR	TCCTCTGACTTCAACAGCGA	GGGTCTTACTCCTTGGAGGC
KLF4 (SeV-Tg)	RT-PCR	TTCCTGCATGCCAGAGGAGCCC	AATGTATCGAAGGTGCTCAA
MYH7	qRT-PCR	TCGTGCCTGATGACAAACAGGAGT	ATACTCGGTCTCGGCAGTGACTTT
MYH10	Sequencing	GTTTGAGATGTCAGTGTTTG	CAGAAACTTTTATACAACAGTCCC
MYH10	qRT-PCR	AAGGTCGGCCGAGACTATG	GAACGAGCCAGCGAAAGA
NANOG	RT-PCR	TGCAAGAACTCTCCAACATCCT	ATTGCTATTCTTCGGCCAGTT
NRIP1	qRT-PCR	GCCAGAAGATGCACACTTGAC	AAGCTCTGAGCCTCTGCTTTC
OCT4 (SeV-Tg)	RT-PCR	CCCGAAAGAGAAAGCGAACCAG	AATGTATCGAAGGTGCTCAA
OCT4 endogenous	RT-PCR	GACAGGGGGAGGGGAGGAGCTAGG	CTTCCCTCCAACCAGTTGCCCCAAAC
PGC1α	RT-PCR	GACATGTGCAAGCAGGAC	ACCAACCAGAGCAGCACA
PKP2	Sequencing	AAGACTTATGACACAATTACGTG	GTGAATAAACCTAAAACCAAGCGG
PKP2	qRT-PCR	GAGAGAAGCACTTTCACTGACTCC	CTCGCTCCAGAGTCATCTCC
PPARγ	qRT-PCR	TGGCAATTGAATGTCGTGTC	GGAAGAAACCCTTGCATCCT
PPARγ*_p1*	ChIP-PCR	TGGAAAAACTGGAACCCTGACAC	AAGGTAACTCGGTGTTTTTGAGGA
PPARγ*_p2*	ChIP-PCR	ACAGTGCTGAGGGCAAGAAT	CCTGGTACCCCTGCTTTCTG
PRDM16	qRT-PCR	AGGGCAAGGAGCGATACAC	TGTCTGGTGAGATTGGCTGA
REX1	RT-PCR	ACCAGCACACTAGGCAAACC	TTCTGTTCACACAGGCTCCA
SEV-TG	RT-PCR	GGATCACTAGGTGATATCGAGC	ACCAGACAAGAGTTTAAGAGATATGTATC
SOX2 (SeV-Tg)	qRT-PCR	ATGCACCGCTACGACGTGAGCGC	AATGTATCGAAGGTGCTCAA
SOX2 endogenous	RT-PCR	GGGAAATGGGAGGGGTGCAAAAGAGG	TTGCGTGAGTGTGGATGGGATTGGTG
TCF21	qRT-PCR	CCCGAGAGTGACCTGAAAGA	GCTCCAGGTACCAAACTCCA
TMEM26	qRT-PCR	ATGGAGGGACTGGTCTTCCTT	CTTCACCTCGGTCACTCGC
TNNT2	qRT-PCR	AGCATCTATAACTTGGAGGCAGAG	TGGAGACTTTCTGGTTATCGTTG
UCP1	qRT-PCR	AGGTCCAAGGTGAATGCCC	GCGGTGATTGTTCCCAGGA
WT1	qRT-PCR	TACACACGCACGGTGTCTTC	GGGCGTTTCTCACTGGTCT

Mouse primers

Gene	Use	Primer forward	Primer reverse
GAPDH	RT-PCR	GCAGTGGCAAAGTGGAGATTG	AGAGATGATGACCCTTTTGGCTCC
Isl1	RT-PCR	GCTATTCACCAATTGTCCAACC	AACCAACACACAGGGAAATCA

Nkx2-5	RT-PCR	CAAGTGCTCTCCTGCTTTCC	GTCCAGCTCCACTGCCTTC
Wt1	RT-PCR	AGTTCCCCAACCATTCCTTC	TTCAAGCTGGGAGGTCATTT

Mouse Taqman probes

Gene	Use	Probe
Gapdh	qRT-PCR	Mm999 999 15_g1 (Thermo Fisher Scientific)
Gata4	qRT-PCR	Mm004 846 89_m1 (Thermo Fisher Scientific)
Isl1	qRT-PCR	Mm006 278 60_m1 (Thermo Fisher Scientific)
Nkx2-5	qRT-PCR	Mm006 577 83_m1 (Thermo Fisher Scientific)

Oligos for shWT1

shWT1	cloning	GAATTAGTCCGCCATCACA
scramble	cloning	GCGCAGGATCGGACTAGAT

Appendix Table S3. Antibodies used in this study

Name of the antibody	Species	Clone	Cat. Nr.	Company	Dilution	Application
Anti-Nanog	rabbit		ab21624	Abcam	1/500	IF
Anti-TRA1-81-Alexa-Fluor-488	mouse		560174	BD	1/20	IF
Anti-SOX17	goat		AF1924	R&D	1/1000	IF
Anti-SOX2	goat		AF2018	R&D	1/1000	IF
Anti-C/EBPα	mouse	G-10	sc-166258	Santa Cruz	1/100	IF
Anti-PPARγ	mouse	E-8	sc-7273	Santa Cruz	1/100	IF
Anti-PPARγ	rabbit	81B8	#2443	Cell Signaling Technology	1/100	IF
Anti-plakophilin-2 (PKP2)	guinea pig		AP09554SU-N	Acris	1/200	IF
Anti-plakophilin-2 (PKP2)	mouse		610788	BD	1/1000	WB
anti-desmoplakin 1/2	mouse		65146	Progen	1/200	IF
Anti-cardiac troponin T (cTNT)	mouse	clone 13-11	MS-295-P1	Thermo Fischer Scientific	1/500	IF
Anti-cardiac troponin T (cTNT)	rabbit	EPR3696	ab92546	Abcam	1/500	IF
Anti-MRTFA	goat	C-19	sc-21558	Santa Cruz	1/100	IF
Anti-Wt1	rabbit		CA1026	Millipore	1/100, 2 µg, 1:500	IF, IP, WB
Anti-pan-cadherin	rabbit		#4068	Cell Signaling Technology	1/1000	WB
Anti-actin	mouse	ACTN05 (C4)	ab3280	Abcam	1/1000	WB
Anti-RhoA	mouse	7F1.E5	ARH94	Cytoskeleton	1/100	IF
Anti-GFP	chicken		GFP-1020	Aves	1/500	IF
Anti-GFP	rabbit		SP3005P	Acris	1/300	IF
Anti-RFP	rat	5F8	5f8	Chromo Tek	1/1000	IF
Anti-alpha skeletal muscle actin	mouse		ab28052	Abcam	1/100	IF
Anti-α-smooth muscle actin (α-sma)	mouse	1A4	A2547	Sigma Aldrich	1/400	IF
Anti-Isl1	mouse	39.4D5	39.4D5	DSHB	1/100	IF
Anti-perilipin 1(Plin1)	rabbit		P1998	Sigma Aldrich	1/500	IF
Anti-non-muscle myosin IIB (MYH10)	mouse	3H2	ab684	Abcam	1/100, 1/2000	IF, WB
Anti-Vimentin	chicken		ab24525	Abcam	1/500	IF
Anti-HA-tag	rabbit		ab9110	Abcam	2 µg	IP

Anti-mouse IgG (HRP)	donkey		715-035-151	Jackson ImmunoResearch	1:10000	WB
Anti-rabbit IgG (HRP)	goat		111-035-144	Jackson ImmunoResearch	1:10000	WB
Anti-mouse IgG light chain (HRP)	rabbit	D3V2A	58802	Cell Signaling	1:1000	WB
Anti-rabbit IgG heavy chain (HRP)	mouse	2A9	ab99702	Abcam	1:1000	WB
Rabbit IgG	rabbit		ab27478	Abcam	2 μg	IP

APPENDIX REFERENCES

Beffagna G, Occhi G, Nava A, Vitiello L, Ditadi A, Basso C, Bauce B, Carraro G, Thiene G, Towbin JA, Danieli GA, Rampazzo A (2005) Regulatory mutations in transforming growth factor-beta3 gene cause arrhythmogenic right ventricular cardiomyopathy type 1. *Cardiovasc Res* 65: 366-73

Erkapic D, Neumann T, Schmitt J, Sperzel J, Berkowitsch A, Kuniss M, Hamm CW, Pitschner HF (2008) Electrical storm in a patient with arrhythmogenic right ventricular cardiomyopathy and SCN5A mutation. *Europace* 10: 884-7

Gerull B, Heuser A, Wichter T, Paul M, Basson CT, McDermott DA, Lerman BB, Markowitz SM, Ellinor PT, MacRae CA, Peters S, Grossmann KS, Drenckhahn J, Michely B, Sasse-Klaassen S, Birchmeier W, Dietz R, Breithardt G, Schulze-Bahr E, Thierfelder L (2004) Mutations in the desmosomal protein plakophilin-2 are common in arrhythmogenic right ventricular cardiomyopathy. *Nat Genet* 36: 1162-4

Klauke B, Kossmann S, Gaertner A, Brand K, Stork I, Brodehl A, Dieding M, Walhorn V, Anselmetti D, Gerdes D, Bohms B, Schulz U, Zu Knyphausen E, Vorgerd M, Gummert J, Milting H (2010) De novo desmin-mutation N116S is associated with arrhythmogenic right ventricular cardiomyopathy. *Hum Mol Genet* 19: 4595-607

Marcus FI, McKenna WJ, Sherrill D, Basso C, Bauce B, Bluemke DA, Calkins H, Corrado D, Cox MG, Daubert JP, Fontaine G, Gear K, Hauer R, Nava A, Picard MH, Protonotarios N, Saffitz JE, Sanborn DM, Steinberg JS, Tandri H et al. (2010) Diagnosis of arrhythmogenic right ventricular cardiomyopathy/dysplasia: proposed modification of the task force criteria. *Circulation* 121: 1533-41

Mayosi BM, Fish M, Shaboodien G, Mastantuono E, Kraus S, Wieland T, Kotta MC, Chin A, Laing N, Ntusi NB, Chong M, Horsfall C, Pimstone SN, Gentilini D, Parati G, Strom TM, Meitinger T, Pare G, Schwartz PJ, Crotti L (2017) Identification of Cadherin 2 (CDH2) Mutations in Arrhythmogenic Right Ventricular Cardiomyopathy. *Circ Cardiovasc Genet* 10

McKoy G, Protonotarios N, Crosby A, Tsatsopoulou A, Anastasakis A, Coonar A, Norman M, Baboonian C, Jeffery S, McKenna WJ (2000) Identification of a deletion in plakoglobin in arrhythmogenic right ventricular cardiomyopathy with palmoplantar keratoderma and woolly hair (Naxos disease). *Lancet* 355: 2119-24

Merner ND, Hodgkinson KA, Haywood AF, Connors S, French VM, Drenckhahn JD, Kupprion C, Ramadanova K, Thierfelder L, McKenna W, Gallagher B, Morris-Larkin L, Bassett AS, Parfrey PS, Young TL (2008) Arrhythmogenic right ventricular cardiomyopathy type 5 is a fully penetrant, lethal arrhythmic disorder caused by a missense mutation in the TMEM43 gene. *Am J Hum Genet* 82: 809-21

Pilichou K, Nava A, Basso C, Beffagna G, Bauce B, Lorenzon A, Frigo G, Vettori A, Valente M, Towbin J, Thiene G, Danieli GA, Rampazzo A (2006) Mutations in desmoglein-2 gene are associated with arrhythmogenic right ventricular cardiomyopathy. *Circulation* 113: 1171-9

Quarta G, Syrris P, Ashworth M, Jenkins S, Zuborne Alapi K, Morgan J, Muir A, Pantazis A, McKenna WJ, Elliott PM (2012) Mutations in the Lamin A/C gene mimic arrhythmogenic right ventricular cardiomyopathy. *Eur Heart J* 33: 1128-36

Rampazzo A, Nava A, Malacrida S, Beffagna G, Bauce B, Rossi V, Zimbello R, Simionati B, Basso C, Thiene G, Towbin JA, Danieli GA (2002) Mutation in human desmoplakin domain binding to plakoglobin causes a dominant form of arrhythmogenic right ventricular cardiomyopathy. *Am J Hum Genet* 71: 1200-6

Syrris P, Ward D, Evans A, Asimaki A, Gandjbakhch E, Sen-Chowdhry S, McKenna WJ (2006) Arrhythmogenic right ventricular dysplasia/cardiomyopathy associated with mutations in the desmosomal gene desmocollin-2. *Am J Hum Genet* 79: 978-84

Taylor M, Graw S, Sinagra G, Barnes C, Slavov D, Brun F, Pinamonti B, Salcedo EE, Sauer W, Pyxaras S, Anderson B, Simon B, Bogomolovas J, Labeit S, Granzier H, Mestroni L (2011) Genetic variation in titin in arrhythmogenic right ventricular cardiomyopathy-overlap syndromes. *Circulation* 124: 876-85

Tiso N, Stephan DA, Nava A, Bagattin A, Devaney JM, Stanchi F, Larderet G, Brahmbhatt B, Brown K, Bauce B, Muriago M, Basso C, Thiene G, Danieli GA, Rampazzo A (2001) Identification of mutations in the cardiac ryanodine receptor gene in families affected with arrhythmogenic right ventricular cardiomyopathy type 2 (ARVD2). *Hum Mol Genet* 10: 189-94

van der Zwaag PA, van Rijsingen IA, Asimaki A, Jongbloed JD, van Veldhuisen DJ, Wiesfeld AC, Cox MG, van Lochem LT, de Boer RA, Hofstra RM, Christiaans I, van Spaendonck-Zwarts KY, Lekanne dit Deprez RH, Judge DP, Calkins H, Suurmeijer AJ, Hauer RN, Saffitz JE, Wilde AA, van den Berg MP et al. (2012) Phospholamban R14del mutation in patients diagnosed with dilated cardiomyopathy or arrhythmogenic

right ventricular cardiomyopathy: evidence supporting the concept of arrhythmogenic cardiomyopathy. *Eur J Heart Fail* 14: 1199-207

van Hengel J, Calore M, Bauce B, Dazzo E, Mazzotti E, De Bortoli M, Lorenzon A, Li Mura IE, Beffagna G, Rigato I, Vleeschouwers M, Tyberghein K, Hulpiau P, van Hamme E, Zaglia T, Corrado D, Basso C, Thiene G, Daliento L, Nava A et al. (2013) Mutations in the area composita protein alphaT-catenin are associated with arrhythmogenic right ventricular cardiomyopathy. *Eur Heart J* 34: 201-10
