# Supplementary material for: Interplay of cell–cell contacts and RhoA/MRTF‐A signaling regulates cardiomyocyte identity
Source: EMBO J. 2018 May 15;37(12):e98133. doi: 10.15252/embj.201798133 (PMC6003642; doi:10.15252/embj.201798133)
Supplement: Supplementary file 2 — Expanded View Figures PDF [file EMBJ-37-e98133-s002.pdf]

Expanded View Figures

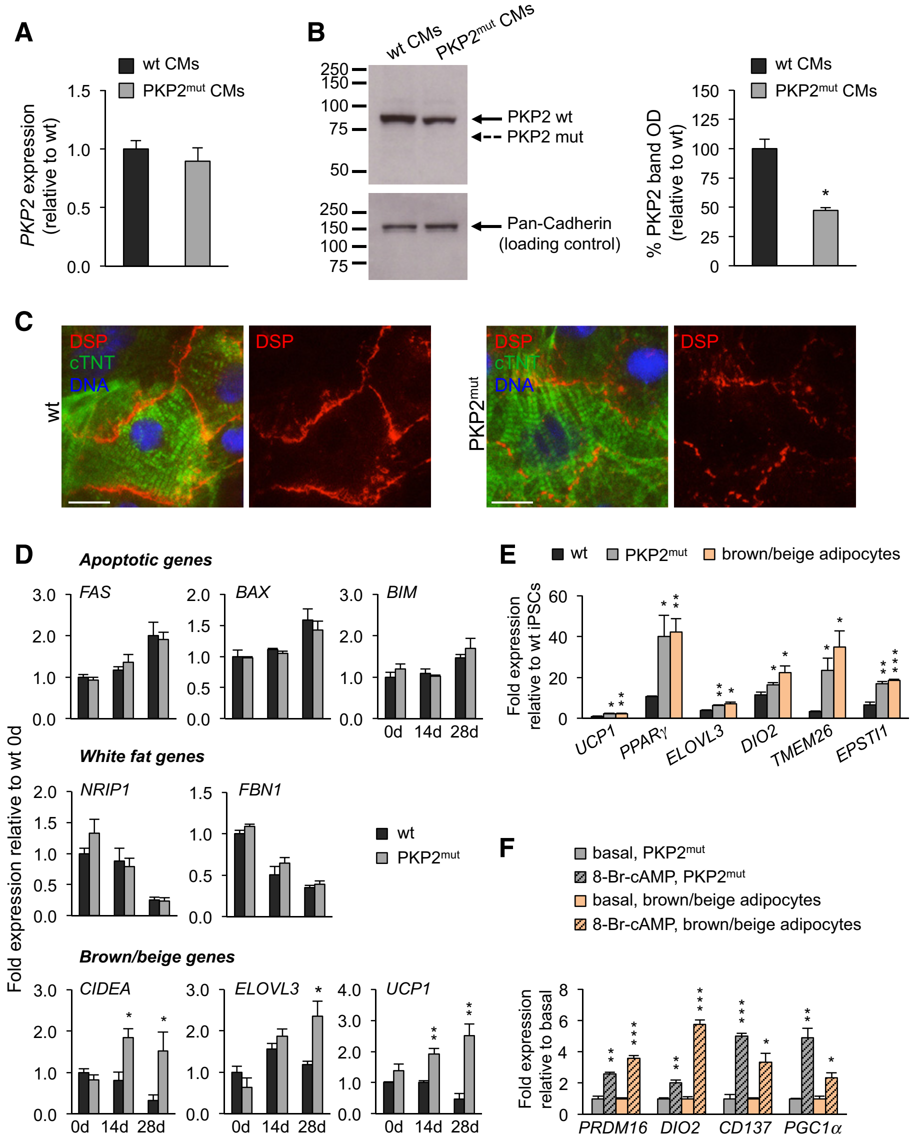

Figure EV1. Expression of desmosomal, pro-apoptotic, and adipocytic genes in PKP2^{mut} CMs.

A qRT–PCR analysis of *PKP2* reveals similar expression levels in wt and PKP2^{mut} CMs ($n = 3$).

B Left, Western blotting using an antibody directed against the N-terminus of PKP2 demonstrates reduced expression of wild-type PKP2 protein (PKP2 wt, 97 kDa) and absence of truncated A587fsX655-PKP2 product (PKP2 mut, 72 kDa) in total lysates of PKP2^{mut} CMs. Pan-Cadherin is shown as a loading control. Right, densitometric readings for PKP2-wt bands reveal an almost 50% reduction in PKP2^{mut} myocytic lysates compared to wt ones. Values are expressed as the integrals (area × mean density) of each band normalized to Pan-Cadherin and relative to wt; $n = 3$; $^{*}P < 0.01$ vs. wt; *t*-test.

C Immunofluorescence analysis indicates an interrupted desmoplakin expression (DSP, red) at the plasma membrane of PKP2^{mut} CMs compared to wt cells. cTNT (green) marks cardiomyocytes. Nuclei are stained with Hoechst 33258 (blue). Scale bars, 12.5 µm.

D qRT–PCR analysis of pro-apoptotic genes and white adipocytic markers shows similar expression levels in wt and PKP2^{mut} CMs over time in culture in adipogenic medium. Brown/beige adipocytic markers were upregulated in PKP2^{mut} compared to wt cells; $n = 3$; $^{*}P < 0.05$, $^{**}P < 0.01$ vs. wt CMs; *t*-test.

E Brown/beige adipocyte-specific genes were similarly upregulated in PKP2^{mut} CMs at 28 days in culture in adipogenic medium and wt iPSC-derived brown/beige adipocytes at day 20 of differentiation compared to wt CMs as determined by qRT–PCR; $n = 3$; $^{*}P < 0.05$, $^{**}P < 0.01$, $^{***}P < 0.001$ vs. wt; *t*-test.

F qRT–PCR analysis of indicated genes shows similar activation in PKP2^{mut} CMs and day-20 iPSC-derived brown/beige adipocytes after treatment with 1 mM 8-Br-cAMP for 48 h. Expression values are relative to basal conditions before treatment in each group; $n = 3$; $^{*}P < 0.05$, $^{**}P < 0.01$, $^{***}P < 0.001$ vs. corresponding PKP2^{mut} CMs or brown/beige adipocytes at basal conditions; *t*-test.

Data information: All data are shown as means ± SEM.

Source data are available online for this figure.

 © 2018 The Authors

A

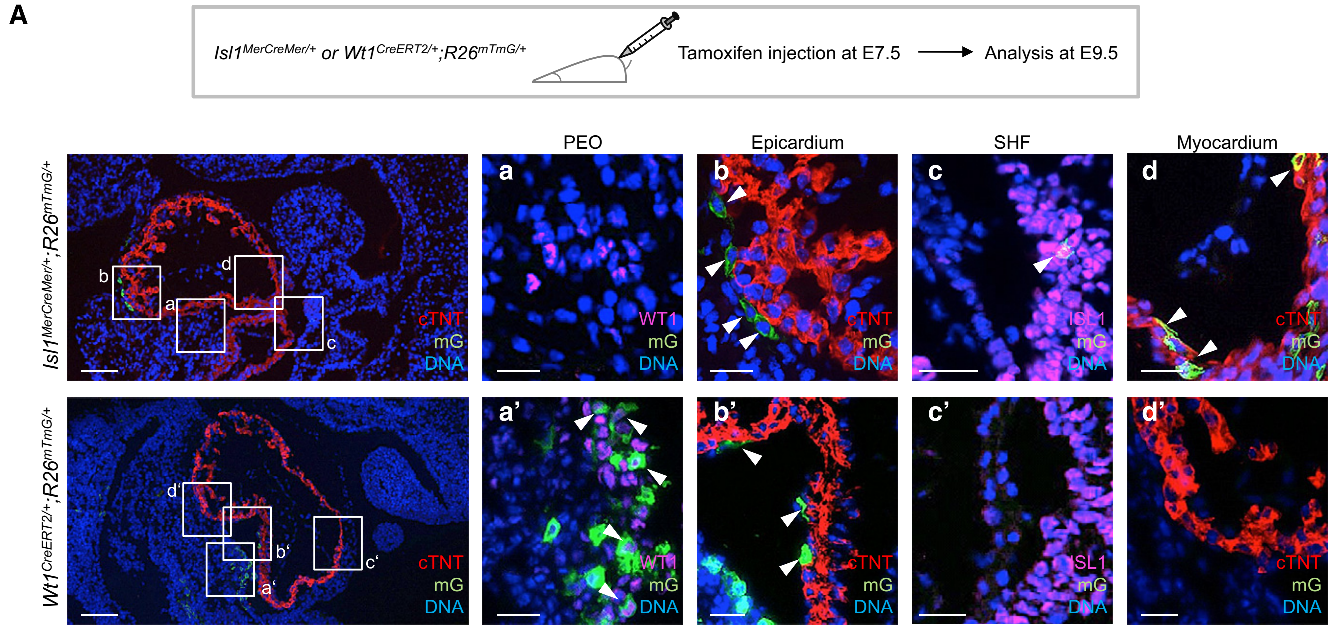

B

Embryo ID	*Isl1*MerCreMer/+;*R26*mTmG/+ (n=6) #749-5	#749-1	#749-3	#715-1	#749-6	#704-3	*Wt1*CreERT2/+;*R26*mTmG/+ (n=6) #197-1	#162-5	#163-5	#188-4	#159-3	#162-7
**PEO**							**1**	**10**	**12**	**16**	**19**	**65**
**Epicardium**					**2**	**5**			**1**	**6**	**1**	**11**
Epi LV					2	5			1	5	1	7
Epi Inflow										1		4
Epi Outflow/RV												
**SHF**	**6**	**18**	**9**	**21**	**25**	**47**						
**Myocytes**	**11**	**7**	**19**	**24**	**22**	**20**						
Outflow/RV	10	6	16	19	18	16						
Inflow	1		3	5	4	4						
LV		1										

C

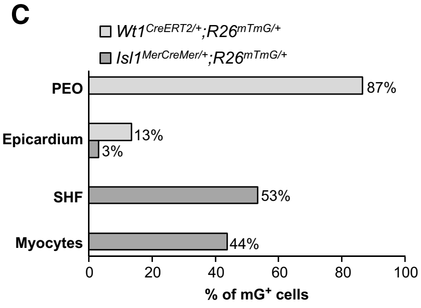

Figure EV2. Localization and quantification of mG-labeled cells in *Isl1*$^{MerCreMer/+}$; *R26*$^{mTmG/+}$ and *Wt1*$^{CreERT2/+}$;*R26*$^{mTmG/+}$ mouse embryos at E9.5.

A Temporal restriction of Cre-mediated labeling of Isl1^{+} and Wt1^{+} progenitors and their derivatives using tamoxifen-inducible *Isl1*$^{MerCreMer/+}$;*R26*$^{mTmG/+}$ and *Wt1*$^{CreERT2/+}$;*R26*$^{mTmG/+}$ mouse lines. Labeling was induced by tamoxifen treatment at E7.5, and embryos were analyzed at E9.5. Immunostaining of cTNT (red), Wt1 (magenta), Isl1 (magenta), and mG (green) in *Isl1*$^{MerCreMer/+}$;*R26*$^{mTmG/+}$ (upper panels) and *Wt1*$^{CreERT2/+}$;*R26*$^{mTmG/+}$ (lower panels). The boxed regions in the left panels are shown in higher magnification (in consecutive sections) in the four right panels a–d and a'–d' for *Isl1*$^{MerCreMer/+}$;*R26*$^{mTmG/+}$ and *Wt1*$^{CreERT2/+}$;*R26*$^{mTmG/+}$, respectively. *Isl1*$^{MerCreMer/+}$-mediated mG labeling was absent in the PEO (a), but it was observed in epicardial cells (b), Isl1^{+} SHF progenitors (c), and CMs (d), as indicated by arrows. *Wt1*$^{CreERT2/+}$-mediated mG labeling was detected in Wt1^{+} cells of the PEO (a') and in epicardial cells (b'), as indicated by arrows; mG expression was absent in Isl1^{+} SHF progenitors (c') and CMs (d'). Scale bars, 100 μm (left panels), 25 μm (right panels).

B Table summarizing the regional distribution and number of mG-labeled cells in *Isl1*$^{MerCreMer/+}$;*R26*$^{mTmG/+}$ and *Wt1*$^{CreERT2/+}$;*R26*$^{mTmG/+}$ mouse embryos at E9.5 as determined in (A). Epi, epicardium; LV, left ventricle; RV, right ventricle.

C Bar graph depicting the percentage of the regional distribution (PEO, epicardium, SHF and myocardium) of mG^{+} cells in *Isl1*$^{MerCreMer/+}$;*R26*$^{mTmG/+}$ and *Wt1*$^{CreERT2/+}$;*R26*$^{mTmG/+}$ mouse embryos at E9.5. Major contribution of Isl1-derivatives was detected in the SHF and myocardium, while most Wt1-derivatives were found in the PEO. Both Isl1- and Wt1-expressing progenitors contributed to the epicardium.

© 2018 The Authors

A

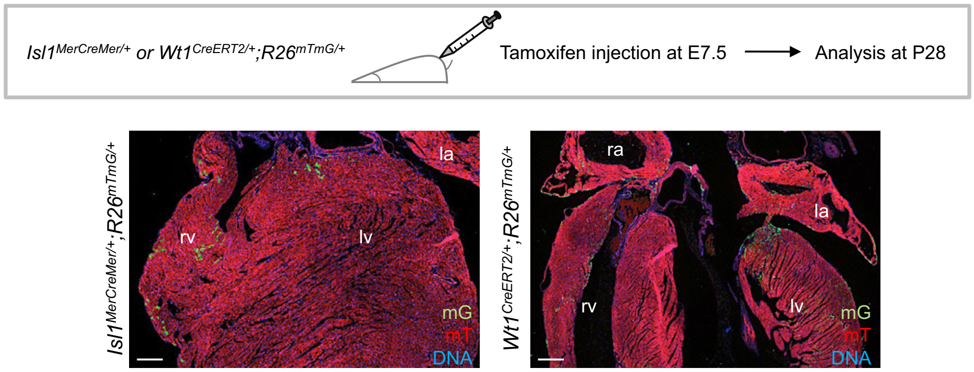

B

Mouse ID	#855-7	#855-4	#770-17	#855-3	#774-11	#770-13	#855-5	#855-1	#797-2	#260-18	#1-21	#482-57	#3-12	#260-15	#2-4	#482-58
	Isl1$^{MerCreMer/+}$;*R26*$^{mTmG/+}$ (n=9)									*Wt1*$^{CreERT2/+}$;*R26*$^{mTmG/+}$ (n=7)						
n°clusters (n°CMs)																
**RA**							**1** (3)	**1** (5)							**1** (>20)	**2** (19;>20)
**RV**		**1** (>20)	**2** (2*>20)	**3** (3;2*>20)	**1** (4)	**2** (6;>20)	**2** (2*>20)	**1** (15)	**6** (2*5;9; 12; 2*>20)							
**LA**	**1** (>20)											**1** (4)	**2** (4;15)	**2** (19;11)		
**LV**				**1** (6)	**1** (6)	**1** (8)	**1** (>20)	**1** (5)			**1** (3)					
**Septum**					**1** (7)	**1** (>20)										**1** (4)
% mG⁺ adipocytes																
**RAV-groove**									31.8	49.8		6.9		14.0	16.1	6.3
**LAV-groove**								46.3		38.0	22.9	16.0	36.0	24.8	14.4	3.6
**Stripe**												18.7	13.8		23.5	4.9

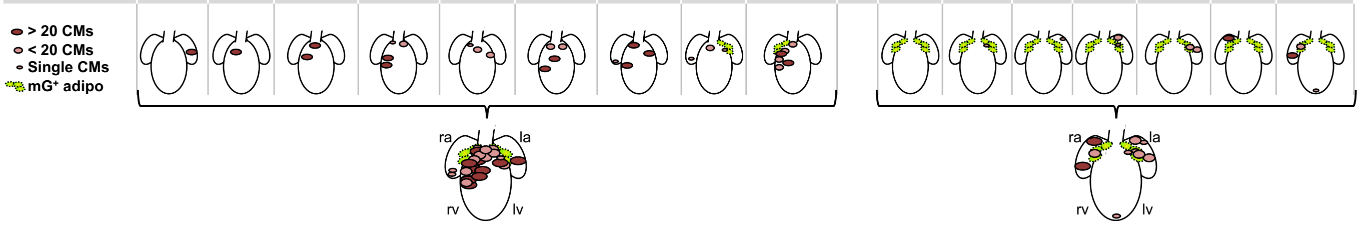

Figure EV3. Localization and quantification of mG-labeled cells in *Isl1*$^{MerCreMer/+}$;*R26*$^{mTmG/+}$ and *Wt1*$^{CreERT2/+}$;*R26*$^{mTmG/+}$ adult mouse hearts at P28.

A Temporal restriction of Cre-mediated labeling of Isl1⁺ and Wt1⁺ progenitors and their derivatives using tamoxifen-inducible *Isl1*$^{MerCreMer/+}$;*R26*$^{mTmG/+}$ and *Wt1*$^{CreERT2/+}$;*R26*$^{mTmG/+}$ mouse lines. Labeling was induced by tamoxifen treatment at E7.5, and adult mouse hearts were analyzed at postnatal day 28 (P28). Immunostaining of mG (green) and mT (red) of *Isl1*$^{MerCreMer/+}$;*R26*$^{mTmG/+}$ (left panel) and *Wt1*$^{CreERT2/+}$;*R26*$^{mTmG/+}$ (right panel). Nuclei are stained with Hoechst 33258. Scale bars, 100 μm. la, left atrium; lv, left ventricle; ra, right atrium; rv, right ventricle.

B Table depicting the regional distribution and the amount of the mG-labeled CMs (as number of clusters and CM number/cluster) and adipocytes (as percentage of total AV groove adipocytes) in *Isl1*$^{MerCreMer/+}$;*R26*$^{mTmG/+}$ and *Wt1*$^{CreERT2/+}$;*R26*$^{mTmG/+}$ adult mouse hearts at P28. Shown are schematic representations of the distribution of mG-expressing cells per each analyzed heart and as a summarized overview for each line. Size of CM clusters is illustrated. LA, left atrium; LAV, left atrioventricular; LV, left ventricle; RA, right atrium; RAV, right atrioventricular; RV, right ventricle.

© 2018 The Authors

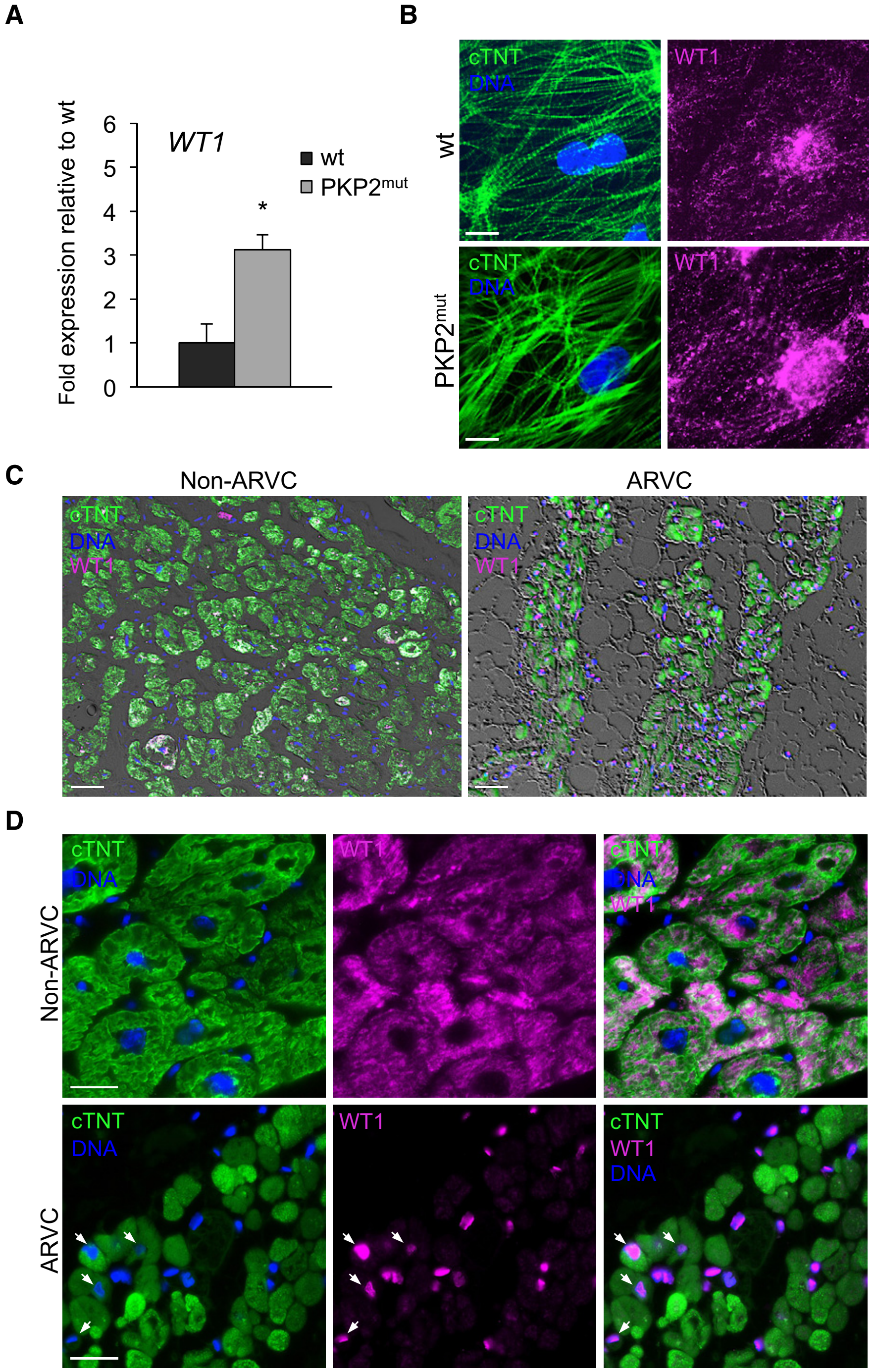

Figure EV4. Upregulated expression and aberrant subcellular localization of WT1 in iPSC-derived PKP2^{mut} CMs and native cardiac muscle cells from ARVC patient heart samples.

A qRT–PCR analysis of *WT1* reveals elevated expression levels in iPSC-derived PKP2^{mut} compared to wt CMs; $n = 3$; $*P < 0.05$ vs. wt; *t*-test. Data are shown as means ± SEM.

B Immunostaining of cTNT (green) and WT1 (magenta) in wt and PKP2^{mut} iPSC-derived CMs. Note the filamentous-like pattern of WT1 cytosolic expression. Nuclei are stained with Hoechst 33258 (blue). Scale bars, 25 μm.

C, D Analysis of adult human myocardium from patients affected and non-affected by ARVC after immunostaining for cTNT (green) and WT1 (magenta). Nuclei are stained with Hoechst 33258 (blue). Low magnification of phase-contrast images merged with immunoflourescence signals are shown in (C). Note intramyocardial fat infiltrations in ARVC conditions. Scale bars, 50 μm. High magnification images demonstrate nuclear localization of WT1 in CMs of ARVC patients (arrows), whereas in non-ARVC individuals only cytosolic WT1 could be detected (D). Scale bars, 25 μm.

Source data are available online for this figure.

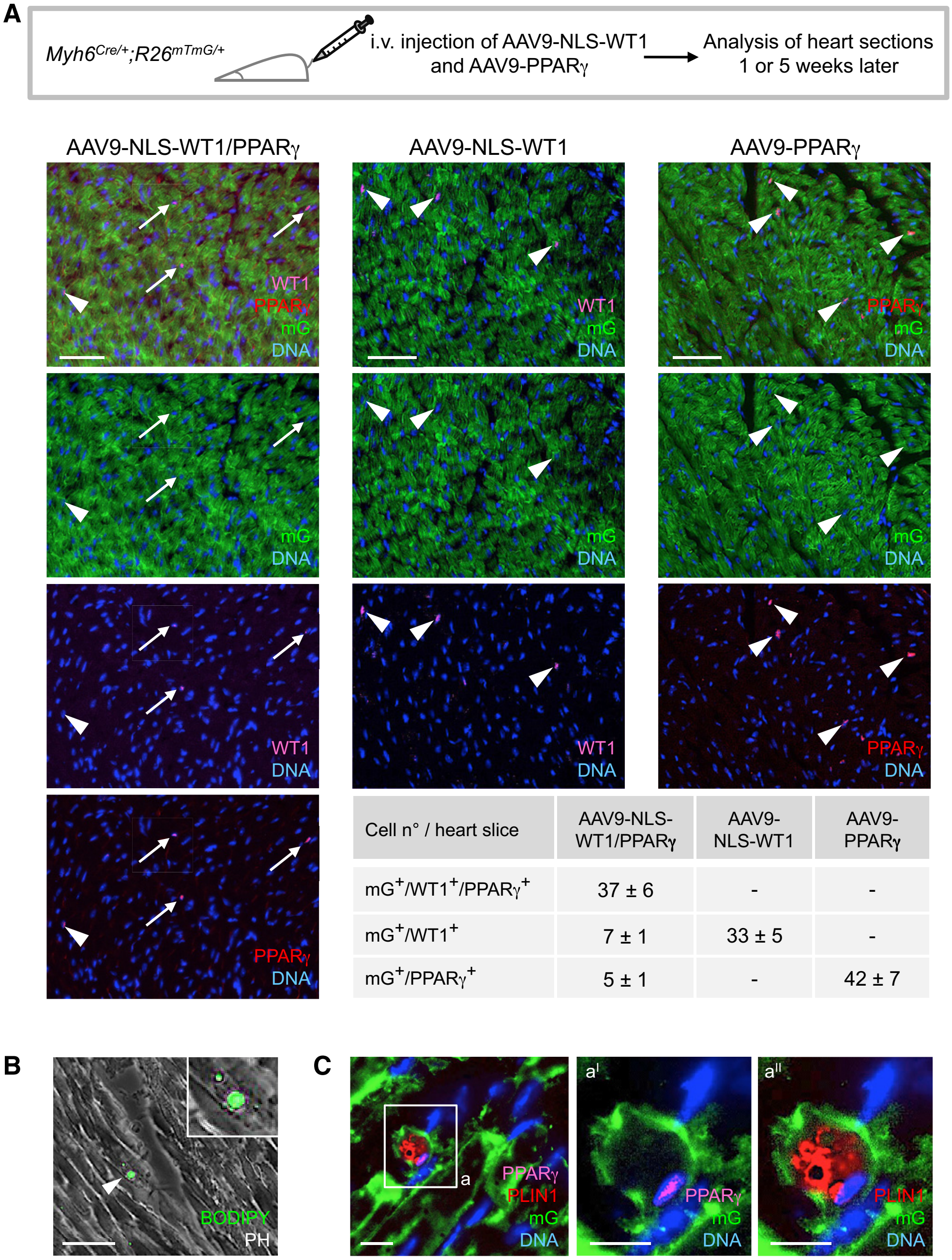

Cell n° / heart slice	AAV9-NLS-WT1/PPARγ	AAV9-NLS-WT1	AAV9-PPARγ
$mG^+/WT1^+/PPAR\gamma^+$	37 ± 6	-	-
$mG^+/WT1^+$	7 ± 1	33 ± 5	-
$mG^+/PPAR\gamma^+$	5 ± 1	-	42 ± 7

Figure EV5. Forced expression of PPARγ and WT1 drives adipocytic conversion of mouse adult CMs *in vivo*.

A Scheme of experimental setup for injection of adeno-associated virus serotype 9 (AAV9) encoding NLS-WT1 (AAV9-NLS-WT1) and PPARγ (AAV9-NLS- PPARγ) in *Myh6*$^{Cre/+}$;*R26*$^{mTmG/+}$ mice and their analysis after 1 or 5 weeks. 2.5 × 10^{12} virus particles were injected intravenously via tail vein. Representative immunostainings of heart sections after 1-week injection of each virus alone or in combination show CMs expressing the lineage marker mG (green) and the Wt1 (cyan) and PPARγ (red) transgenes. Nuclei are stained with Hoechst 33258. Arrowheads and arrows indicate mG^+ CMs infected with one or both viruses, respectively. Scale bars, 50 μm. Quantification of transgene expressing mG^+ CMs reveals high rate of co-transduction. Three random heart slices per mouse and three mice per virus condition were analyzed.

B, C Analysis of heart sections 5 weeks after infection with AAV9-NLS-WT1 and AAV9-PPARγ. (B) shows a representative phase-contrast (PH) image merged with BODIPY fluorescence signal (green) visualizing a lipid-filled cell within the myocardium of a mouse that received both viruses. Higher magnification is shown in the inset. Scale bar, 50 μm. Note that $BODIPY^+$ cells with enlarged multilocular lipid droplets were detected exclusively in the heart of mice infected with both AAV9-NLS-WT1 and AAV9-PPARγ at a frequency of 3–5 cells per heart section. Arrowhead indicates a $BODIPY^+$ cell. In (C), subsequent immunofluorescence detection of mG (green), PPARγ (magenta), and the adipocyte marker PLIN1 (red) ultimately identifies infected CMs that underwent adipocytic conversion solely in mice treated with both viruses. Scale bar, 10 μm. The boxed region is shown in higher magnification (panels a^{I} and a^{II}). Scale bars, 25 μm.

Source data are available online for this figure.

 © 2018 The Authors
