# Supplementary figures and images for: Interplay of cell–cell contacts and RhoA/MRTF‐A signaling regulates cardiomyocyte identity
Source: EMBO J. 2018 May 15;37(12):e98133. doi: 10.15252/embj.201798133 (PMC6003642; doi:10.15252/embj.201798133)

Data source for Fig. S6B

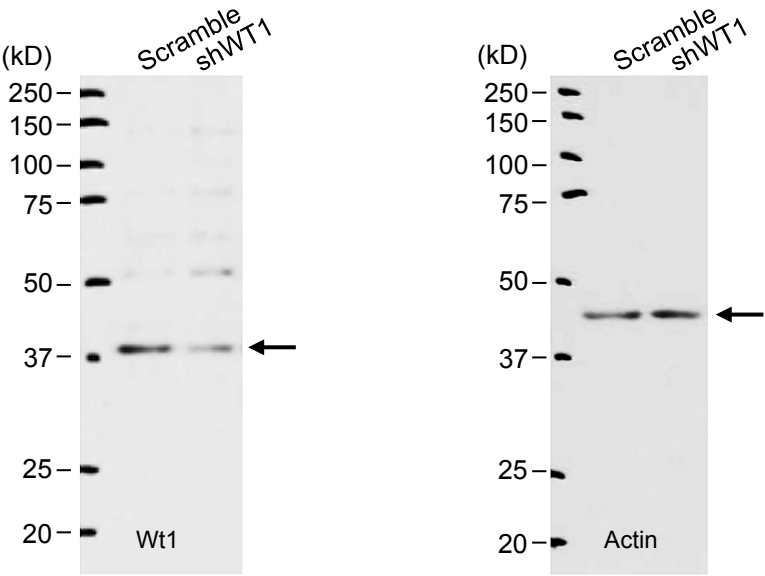

Supplement: Supplementary file 3 — Source Data for Expanded View and Appendix [file EMBJ-37-e98133-s003.zip › Source_Data_Appendix_figures/EMBOJ-2017-98133R_SourceDataForFigureS6b.pdf]

Data source for Fig. S8I

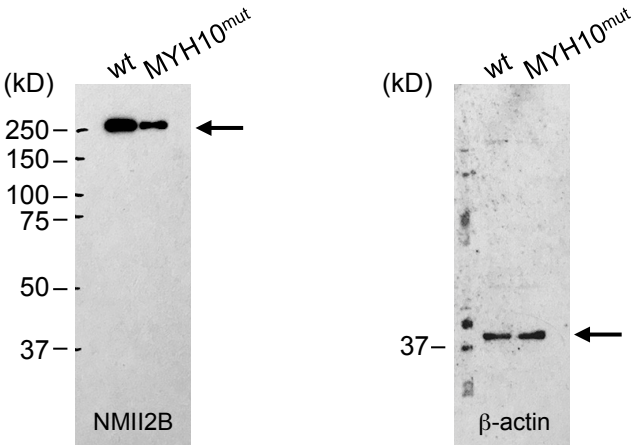

Supplement: Supplementary file 3 — Source Data for Expanded View and Appendix [file EMBJ-37-e98133-s003.zip › Source_Data_Appendix_figures/EMBOJ-2017-98133R_SourceDataForFigureS8i.pdf]

Data source for EV1B

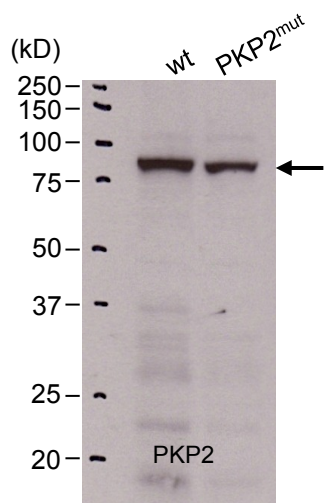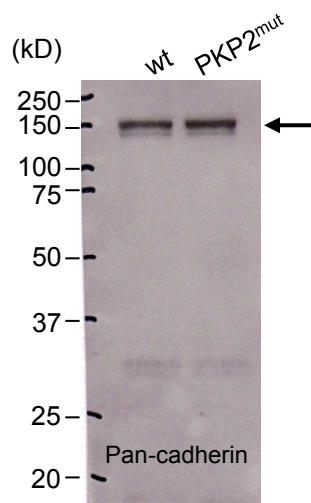

Supplement: Supplementary file 3 — Source Data for Expanded View and Appendix [file EMBJ-37-e98133-s003.zip › Source_Data_EV_figures/EMBOJ-2017-98133R_SourceDataForFigureEV1b.pdf]

Data source for Fig. 3H

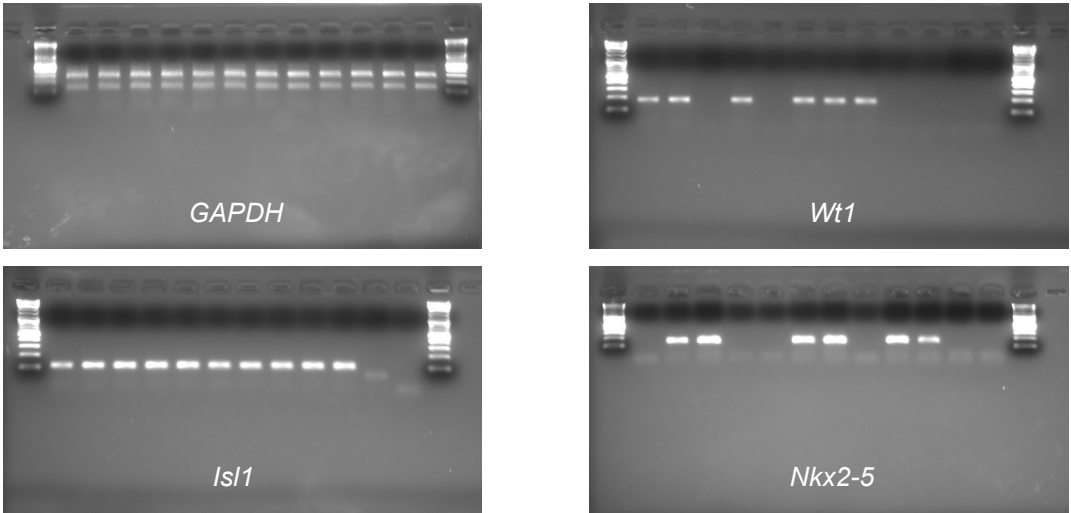

Supplement: Supplementary file 6 — Source Data for Figure 3 [file EMBJ-37-e98133-s005.zip › Source_data_Fig_3H.pdf]

Data source for Fig. 4I

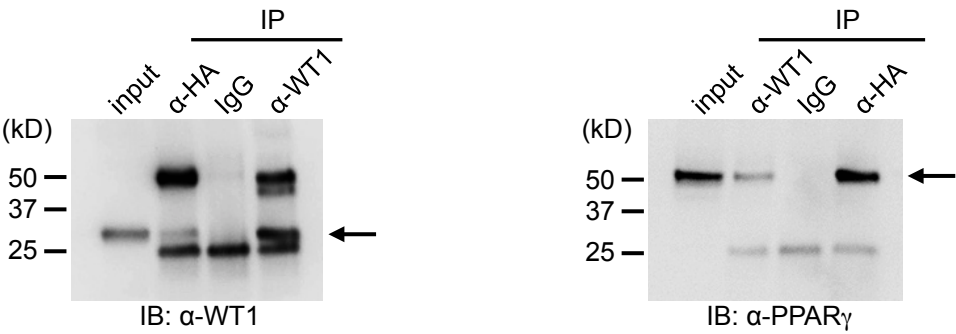

(kD)

Supplement: Supplementary file 7 — Source Data for Figure 4 [file EMBJ-37-e98133-s006.zip › Source_data_Fig_4I.pdf]
